# Metallothionein family genes in kiwifruit: characterization and determining their roles in plant’s response to different stresses

**DOI:** 10.3389/fpls.2024.1425072

**Published:** 2024-10-18

**Authors:** Linya Liu, Shuyi Song, Na Yang, Bin He, Lusheng Xin, Yacheng Huang

**Affiliations:** ^1^ School of Biological Science and Technology, Liupanshui Normal University, Liupanshui, China; ^2^ School of Public Health, Jining Medical University, Jining, China

**Keywords:** kiwifruit, regulation, AcMT genes, ROS, stress response

## Abstract

Kiwifruit growth and development are severely affected by various biotic and abiotic stresses, especially cold stress and the bacterial disease caused by *Pseudomonas syringae* pv. *actinidiae* (Psa). Metallothioneins (MTs) are a group of cysteine-rich proteins that play crucial roles in stress response, metal detoxification, and homeostasis in plants. However, the protective role of these MTs in kiwifruit remains to be elucidated. In the present study, four *AcMT* genes were identified in the Hongyang kiwifruit genome, namely, two Type 2 isoforms (*AcMT2* and *AcMT2a*) and two Type 3 isoforms (*AcMT3a* and *AcMT3b*) located separately on four different chromosomes. The hormones and stress response *cis*-elements within the promoter regions of these *AcMTs* were characterized. It was revealed that the four *AcMT* genes exhibited different expression patterns in different tissues: *AcMT2* and *AcMT2a* were expressed at much higher levels in the fruit, male flower, female flower, root, and bark, while *AcMT3a* was expressed mainly in the fruit and *AcMT3b* was expressed highly in the bark. The expression patterns of these *AcMT* genes after exposure to Psa infection and different phytohormones, including gibberellic acid A3(GA_3_), ethylene (ET), and abscisic acid (ABA), were evaluated. It was revealed that in response to Psa infection, the main *AcMTs* in each tissue (those with expression levels higher compared to the other *MTs* in that tissue) were downregulated during the early stage in kiwifruits, followed by a recovery phase. In addition, most *AcMTs* were downregulated after exposure to ET and GA_3_, while type 2 *AcMTs* (*AcMT2* and *AcMT2a*) were upregulated after treatment with ABA. The overexpression of *AcMTs* in *Escherichia coli* presented a higher tolerance to H_2_O_2_, heavy metals, low temperature, and high temperature. Collectively, these findings demonstrated the protective roles of *AcMTs* in terms of stress resistance conferred through plant hormone-related signal pathways.

## 1 Introduction

Metallothioneins (MTs) are a group of small, cysteine-rich proteins with a high binding affinity for metal ions, particularly the ones toxic to cells, such as cadmium (Cd), lead (Pb), copper (Cd), and mercury (Hg) (Hassinen et al., 2011). MT proteins were first identified in horse kidneys, following which studies revealed that *MT* genes are conserved across various organisms, including plants, animals, bacteria, and fungi (Cobbett and Goldsbrough, 2002; Zhou et al., 2006; Leszczyszyn et al., 2013; Barbosa et al., 2017). In plants, MTs are classified into four types based on the distribution of Cys residues in these proteins, and these different types of MTs reportedly exhibit distinct tissue distribution with potentially diverse functions (Usha et al., 2009; Freisinger, 2011). In *Arabidopsis*, seven MT genes were characterized and categorized into four types: *AtMT1a* and *AtMT1c* in Type 1 MT, *AtMT2a* and *AtMT2b* in Type 2 MT, *AtMT3* in Type 3 MT, and *AtMT4a* and *AtMT4b* in Type 4 MT. These four types of MTs were reportedly expressed predominantly in the roots, leaves, fruits, and seeds, respectively (Ledger and Gardner, 1994; Zhou and Goldsbrough, 1994; Hsieh et al., 1995; Zhou and Goldsbrough, 1995; Hsieh et al., 1996). The roles of MTs in plant’s responses to various environmental signals and developmental processes have been studied extensively in recent studies, which have revealed that the expressions of MTs were precisely regulated in response to different stresses (Leszczyszyn et al., 2013; Benatti et al., 2014; Barbosa et al., 2017). MTs play essential roles in plant survival, development, and adaptation to changing environmental conditions. The presence of different motifs in the promoters of MT genes implies that these genes are important for the regulation and execution of a normal developmental process and stress responses in plants (Cheng et al., 2021; Gao et al., 2022).

The roles of MT genes, specifically in plant stress tolerance, are multifaceted. MTs were initially studied because of their abundant Cys residues, which could reversibly bind toxic and essential metal ions, enabling MTs to play crucial roles in the homeostasis and detoxification of metal ions in cells (Hamer, 1986; Hassinen et al., 2009). This functional ability of MTs to sequester toxic metals and thereby prevent toxicity in various processes occurring within the cells is crucial for plant stress tolerance, especially in environments with high metal concentrations. MT overexpression in different model systems, including *Arabidopsis*, tobacco, yeast, and *Escherichia coli*, has been demonstrated to contribute to the metal tolerance and homeostasis mechanisms. Specifically, the overexpression of HbMT2 in *E. coli* reportedly enhanced the tolerance of these bacteria to heavy metals (Zn^2+^ and Cu^2+^) (Huang et al., 2018). The overexpression of CrMT in *Canavalia rosea* enhanced the tolerance of this yeast to heavy metals owing to the metal-chelating ability of the CrMT that was overexpressed in the yeast cells (Zou et al., 2022). The overexpression of the tobacco MT gene *NtMT2F* in *E. coli* and *Arabidopsis thaliana* reportedly enhanced tolerance to Cd stress (Li et al., 2023). In addition to metal-binding properties, scavenging of reactive oxygen species (ROS) is conferred to the MTs due to the abundance of Cys residues (Hassinen et al., 2011; Lv et al., 2013). This enables the MTs to play a protective role against oxidative stress. ROS accumulation occurs during multiple stress conditions, such as drought and exposure to UV radiation and various pollutants. MTs scavenge the ROS and thereby assist in maintaining the redox balance in cells, contributing to cellular resilience. An increasing number of researchers have begun investigating the roles of genes encoding those MTs that serve as ROS scavengers in various physiological processes to understand the contribution of these genes to plant defense responses against different biotic and abiotic stresses (Huang et al., 2018; Zou et al., 2022); cell growth, proliferation, and regulation (Grennan, 2011); root development (Yuan et al., 2008); fruit ripening (Moyle et al., 2005); and senescence (Guo et al., 2003). Transgenic plants with OsMT2b overexpression reportedly exhibited increased vulnerability to bacterial blight and blast fungus due to a sharp decline in the generated ROS (Wong et al., 2004). SbMT-2 overexpression in tobacco significantly improved the tolerance to salt, osmotic, and metal stresses in transgenic tobacco plants (Ahn et al., 2012). In addition, salinity stress reportedly induced the transcriptional activation of *OsMT1e-P*, the overexpression of which enhanced the tolerance of tobacco to salinity, drought, cold, and heat stresses (Kumar et al., 2012). Collectively, the above findings related to different *MT* genes from various plants suggest that MTs improve stress tolerance in plants through their ROS-scavenging function.

Kiwifruits have a high nutritional and commercial value, and are, therefore, cultivated widely across different parts of the world, including New Zealand, Italy, Chile, Iran, and China (Ferguson, 2014). “Hongyang” kiwifruit, derived from *Actinidia chinensis* var. *chinensis* (Li et al., 2017), is becoming a recent favorite among people owing to its delicious taste, unique flavor, and high content of beneficial components such as vitamin C, anthocyanins, amino acids, and minerals. However, in their natural habitat, “Hongyang” kiwifruits frequently encounter various biotic and abiotic stresses, especially those related to bacterial diseases (infection with Psa, *Pseudomonas syringae* pv. *actinidiae*), drought, heavy metal exposure, and extreme temperatures, which significantly impacts their growth and development. Previous studies reported that an MT-like protein gene exhibited higher expression during kiwifruit ripening (Ledger and Gardner, 1994) and was also expressed differentially in Psa-infected kiwifruits (Wang et al., 2018). Therefore, considering the above findings and the crucial role of MTs against multiple stresses, the present study was conducted to investigate the roles of kiwifruit MTs in response to Psa infection and other stresses. Four *AcMT* genes were screened out from the “Hongyang” kiwifruit genome and then analyzed for their expression patterns in different tissues and in response to Psa infection and exposure to various phytohormones. Furthermore, the identified *AcMTs* were overexpressed in *E. coli* to investigate their roles in the plant’s response against stresses due to heavy metals, temperature, and ROS, thereby obtaining further insights into the functions of MTs in kiwifruit.

## 2 Materials and methods

### 2.1 Plant materials and treatments

The 8-year-old *A. chinensis* cv. Hongyang selected for the present study was planted (26.28 N and 104.48 E) at the kiwifruit plantation in Shaomi town, Liupanshui City, Guizhou Province, China. In order to clone the MT genes and analyze their tissue distribution, the female flowers, male flowers, leaves, roots, and bark of the plants were harvested at the flowering stage; the fruits were harvested at 118 days after flowering; and the seeds were harvested after the fruit matured and softened. In order to study the effects of hormone treatments on the expressions of the *AcMTs* in the fruits, the fruits harvested at 18 days after flowering were steeped for 3 s with 20 mg/L CPPU, 50 mg/L ethephon (ET, an ethylene-releasing compound), 50 mg/L GA_3_, and 10 mg/L ABA at 0, 3, 6, and 9 h prior to sampling. Fruits from the treated plants were collected at each time point and subjected to RNA extraction, and untreated plants were used as controls. The experiment was repeated three times with three technical replicates (five trees per replicate).

### 2.2 RNA extraction and cDNA synthesis

Total RNA was extracted from kiwifruit tissues as described in a previous study (Liu et al., 2020). First-strand cDNA was then synthesized from the extracted RNA using the RevertAid First Strand cDNA Synthesis Kit (ThermoFisher, Shanghai, China), according to the manual.

### 2.3 Isolation of the *MT* genes in kiwifruit

The sequences of the MT genes identified in other plant species were downloaded from the National Center for Biotechnology Information (NCBI) database and used as query sequences for the basic local alignment search tool (BLAST) analysis of the collective transcriptome database established by our research group and the Kiwifruit Genome Database (http://kiwifruitgenome.org/) (Yue et al., 2020). The full-length cDNA sequences of four *AcMTs* were thus obtained. Gene identification was confirmed through an RT-PCR conducted using the primers provided in **Supplementary Table S1**, followed by DNA sequencing.

### 2.4 Multiple sequence alignment and bioinformatics analysis

The obtained cDNA sequences were submitted to the NCBI database for BLAST searches. The amino acid sequences of the four AcMTs and the MTs of other plant species were aligned using ClustalW (v2.0) software (Larkin et al., 2007). A phylogenetic tree was constructed using the neighbor-joining method in MEGA 7.0 with 1,000 bootstrap replicates. The molecular weights (MWs) and the theoretical isoelectric point values of the AcMTs were calculated using the ProtParam online tool (http://www.expasy.ch/tools/protparam.html). The *AcMT* promoters were characterized by obtaining 2,000-bp genomic regions upstream of the initiation codon (ATG) of each of the four *AcMT* genes from the Kiwifruit Genome Database (http://kiwifruitgenome.org/) (Yue et al., 2020). The *cis*-elements were predicted using Plantcare (http://bioinformatics.psb.ugent.be/webtools/plantcare/html/) (Lescot et al., 2002). TBtools (https://github.com/CJ-Chen/TBtools) was employed to predict gene structures and conserved domains (Chen et al., 2020). The conserved motifs were analyzed using MEME (http://meme-suite.org/) with the following parameters: maximum number, 5; site distribution, any number of repetitions; minimum width, 6; maximum width, 50 (Brown et al., 2013). TBtools was then employed to draw the location figure, and the chromosomal positions of the *AcMT* genes were obtained from the GFF file (Chen et al., 2020). MCScanX, Circos, and Dual Synteny Plotter programs in TBtools were employed to calculate and draw the collinear genes in the kiwifruit genome and among the different species (including *A. thaliana*, *Rosa chinensis*, *Zea mays*, *Musa acuminata*, and *Populus trichocarpa*) (Ma et al., 2023). The KaKs_Calculator2.0 was employed to calculate the *Ka/Ks* value of each gene pair (Wang et al., 2010).

### 2.5 Expressions of the *AcMTs* in response to Psa infection

The effects of bacterial infection on the expressions of the *AcMTs* were evaluated, for which the RNA-seq data (Wang et al., 2017; Michelotti et al., 2018; Song et al., 2019) in the Kiwifruit Genome Database (http://kiwifruitgenome.org/) were downloaded (Yue et al., 2020). The data were from the shoot, leaf, and seedling samples of kiwifruit infected with Psa (datasets PRJNA514180, PRJNA328414, and PRJNA436459, respectively). The raw RNA-seq reads were processed using Trimmomatic to remove adapters and low-quality bases, and the trimmed reads shorter than 80% of the original length were discarded. Gene transcript levels were expressed as the expected number of reads per kilobase of the transcript sequence per million base pairs sequenced (RPKM), and the corresponding heat map was generated using R (v3.4.0) with log_2_^(FC)^. The *p*-values were adjusted using Benjamini and Hochberg’s approach to constrain the false discovery rate (FDR). The genes with an adjusted *p*-value (FDR) of <0.05, as determined using DESeq, were designated as the differentially expressed genes.

### 2.6 The qRT-PCR analysis

The expressions of the *AcMT* genes were determined using the qRT-PCR analysis, performed as described in a previous study (Liu et al., 2017). The primers used in the qRT-PCR are listed in **Supplementary Table S1**. The relative expression levels of the *AcMT* genes were calculated using the 2^−ΔΔCT^ method (Trick et al., 2021).

### 2.7 Expression of the AcMTs in *E. coli*

The coding region of each of the *AcMTs* was amplified through PCR using primer pairs listed in **Supplementary Table S2**. The PCR products were then inserted into the expression vector pET-28a(+) as described by Huang et al. (2018). The recombination proteins were expressed in *E. coli* BL21 under induction with 0.4 mM isopropyl β-D-thiogalactopyranoside (IPTG) at 37°C for 4 h, followed by SDS-PAGE for protein identification (Laemmli, 1970). The tolerance of the transformed *E. coli* BL21 cells to abiotic stress was investigated by comparing the bacterial growth status of the strains containing pET-28a-*AcMTs* with that of the strains harboring the blank vector alone, as described in a previous report (Huang et al., 2018). The strains were subjected to abiotic stresses as described by Huang et al. (2018). Bacterial growth was monitored and quantified by measuring the OD_600_ after incubation at 20°C or 45°C for 0.5, 1.0, 1.5, 2.0, 2.5, 3.0, and 3.5 h.

### 2.8 Statistical analysis

The resulting data were expressed as means ± standard errors (SEs) of three independent biological replicates. Statistical analysis was performed using one-way ANOVA in GraphPad Prism 8.0, and the values indicated using different letters represented significant differences (*p* < 0.05).

## 3 Results

### 3.1 Identification and sequence analysis of *AcMTs*

**Figure 1:**
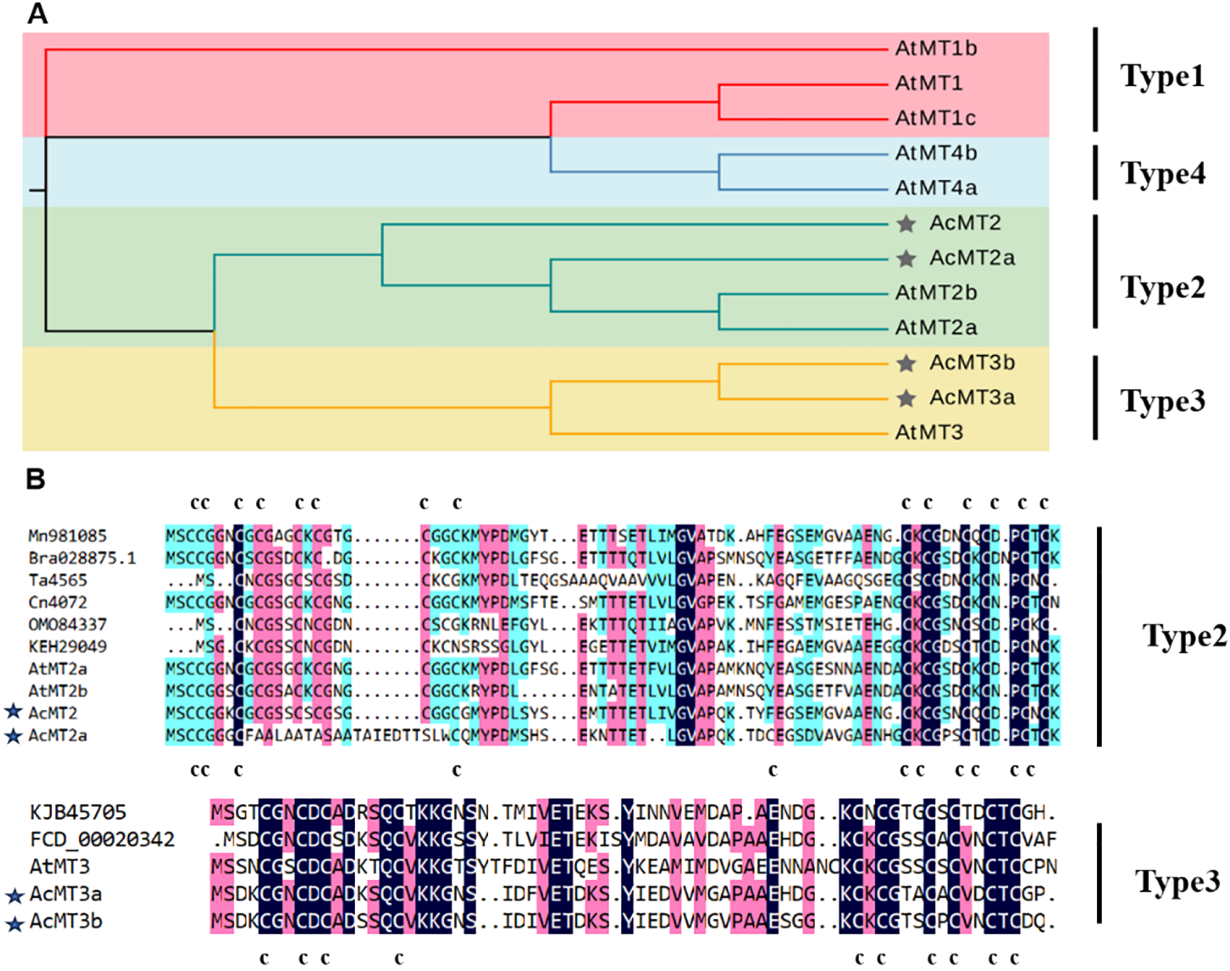
Multiple sequence alignment results, the phylogenetic tree, and the gene structure of kiwifruit MTs. **(A)** Comparison of the deduced amino acids of kiwifruit MTs, along with their homologs from other plant species. The conserved cysteine residues are marked by the letter C. Identical or conserved amino acids are shaded in black, pink, and green. **(B)** The phylogenetic tree for the proteins in kiwifruit and *Arabidopsis* MTs, constructed using MEGA version 6.0. The AcMTs are depicted using a black pentagram.

Four *AcMT*s were identified from the *A. chinensis* cv. Hongyang genome: *AcMT2*, *AcMT2a*, *AcMT3a*, and *AcMT3b.* The identification was based on the characteristics of encoded proteins. These four AcMTs were classified according to the sequences of their corresponding *Arabidopsis* homologs into Type 2 (AcMT2 and AcMT2a) and Type 3 (AcMT3a and AcMT3b) MT proteins in plants (**Figure 1A**). The length of the open reading frame (ORF) of the four *AcMT*s ranged from 195 to 255 base pairs (bps). These ORFs encoded proteins containing 64–84 amino acids (aa) with MWs ranging from 6.6 to 8.5 kDa. The theoretical isoelectric point (p*I*) values of these proteins ranged between 4.38 and 4.61 (**Table 1**). The Cys residue content in the four *AcMT*s ranged from 13.1% to 17.9% (**Table 1**), which is comparable to the range reported for MTs from other plants. AcMT2 contained 14 Cys residues, 8 of which were located in the N-terminus, and 6 were located in the C-terminus, which is characteristic of a typical Type 2 MT protein. AcMT2a, on the other hand, had 11 Cys residues, which were split into four Cys-rich domains by three Cys-free spacers with 25, 21, and 13 aa. Three of the Cys residues were located in the N-terminus, and six were located in the C-terminus. Two Cys-rich domains were located in the middle of the N-terminus. The C-terminus contained one Cys residue. AcMT3a and AcMT3b had 10 Cys residues each, and among the 10 residues in each protein, 4 were located in the N-terminus, and 6 were located in the C-terminus of the protein, which is characteristic of a typical Type 3 MT protein (**Figure 1B**).

**Table 1:** Characteristics of the *AcMT* genes.

Gene name	ORF (bp)	Length (aa)	MW (kDa)^a^	p*I*	Cys number^b^	Cys content (%)	Space^c^ (aa)
*AcMT2* *AcMT2a* *AcMT3a* *AcMT3b*	237 255 195 195	78 84 64 64	7.8 8.5 6.7 6.6	4.49 4.45 4.61 4.38	8 + 6 3 + 1+1 + 6 4 + 6 4 + 6	17.9 13.1 15.6 15.6	40 21/25/13 32 32

^a^MW, molecular weight. ^b^Cys number with (A+B) means A in the N-terminus and B in the C-terminus; (A+B+C) means A in the N-terminus, C in the C-terminus, B in the middle of both the N-terminus and the C-terminus; ^c^the number of aa between Cys clusters.

**Figure 2:**
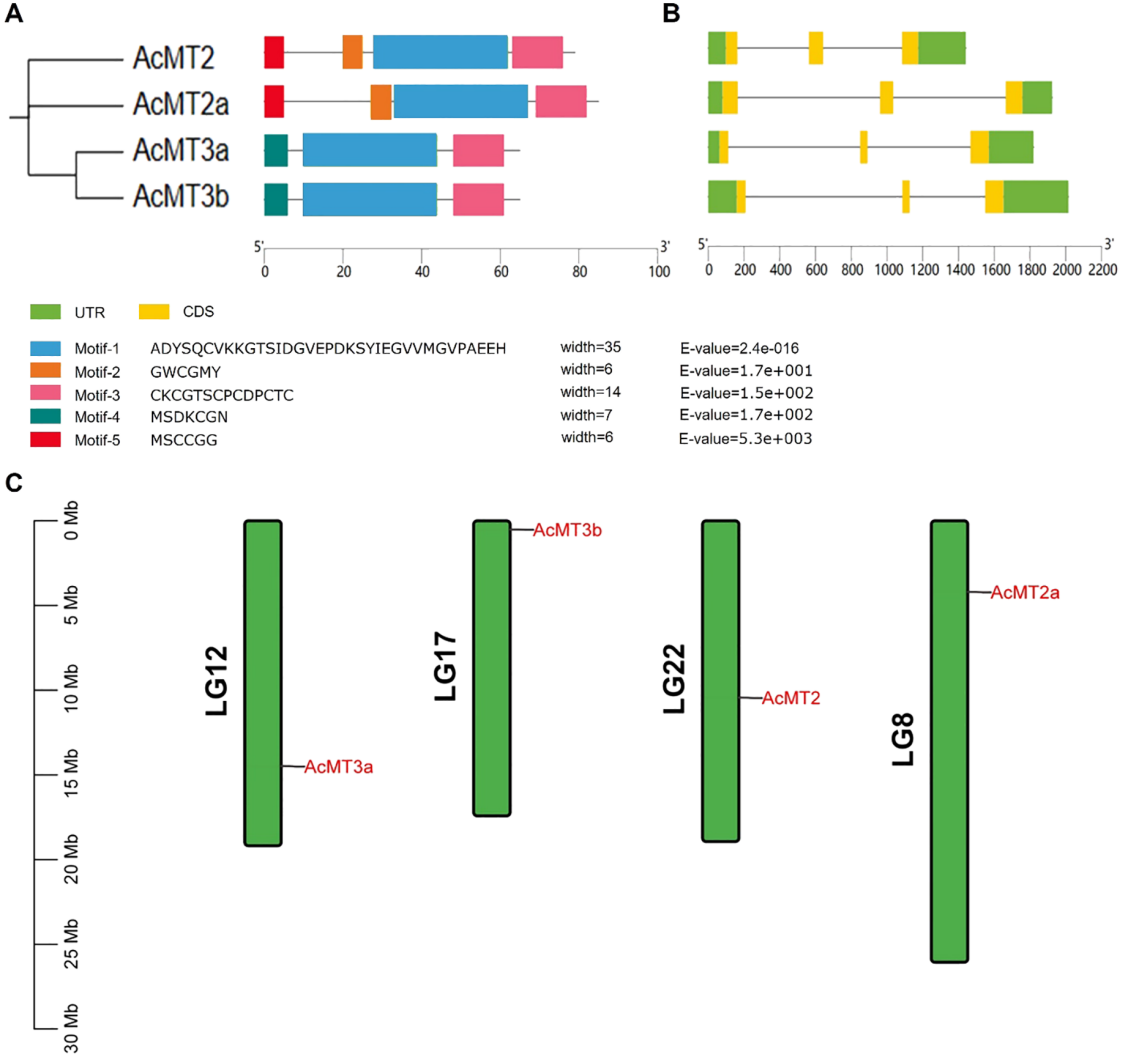
The conserved protein motifs, the gene structure, and the chromosomal distribution of the four *AcMTs* in kiwifruit. **(A)** The conserved protein motifs of the four *AcMT*s. Five patterns of the conserved protein motifs are depicted within different colored boxes. **(B)** The gene structure of the four *AcMT*s. **(C)** The chromosome distribution of the *AcMT* genes, with each of the four *AcMT* genes mapped onto four different chromosomes in kiwifruit.

The structures of the *AcMT* genes were analyzed (**Figure 2B**). It was observed that all *AcMT* genes had a similar exon–intron arrangement. The MEME program was then applied to screen the conserved protein motifs among the AcMTs. The results revealed that each of the AcMTs contained five conserved motifs, among which motif 1 and motif 3 were conserved in all four AcMT proteins (**Figure 2A**). Motifs 1, 3, and 4 were present in Type 3 MTs, while Type 2 MTs contained Motifs 1, 2, 3, and 5. AcMTs of the same type were revealed to share the conserved gene structure and protein domains. The chromosome distribution of the four *AcMTs* is presented in **Figure 2C**. The four *AcMTs* were mapped independently on four different chromosomes. *AcMT2*, *AcMT2a*, *AcMT3a*, and *AcMT3b* were distributed on chromosomes 22, 8, 12, and 17, respectively, each located in the forward direction.

### 3.2 Phylogenetic analysis of the four AcMTs

**Figure 3:**
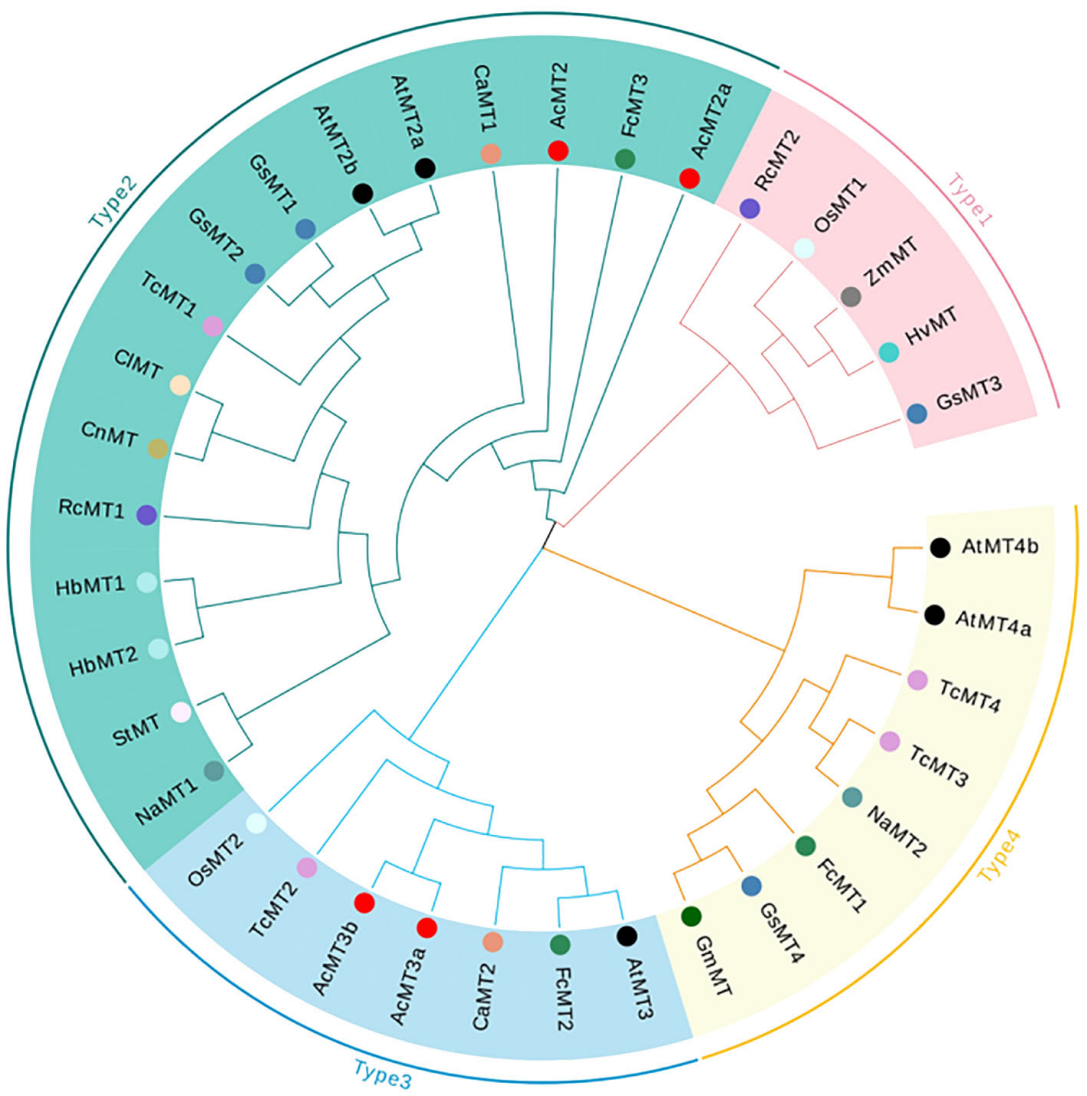
Phylogenetic analysis of 36 *MT* gene-encoding proteins. The blank circles represented the *AcMT*s. The different color circles represent different species. AcMT2, AcMT2a, AcMT3a, and AcMT3b were from *A. chinensis*; AtMT2a, AtMT2b, AtMT3, AtMT4a, and AtMT4b were from *A. thaliana*; CaMT1 and CaMT2 were from *Capsicum annuum*; ClMT was from *Citrullus lanatus*; CnMT was from *Codonopsis lanceolata*; FcMT1, FcMT2, and FcMT3 were from *Ficus carica*; GmMT was from *Glycine max*; GsMT1, GsMT2, GsMT3, and GsMT4 were from *Glycine soja*; HbMT1 and HbMT2 were from *Hevea brasiliensis*; HvMT was from *Hordeum vulgare* subsp. vulgare; NaMT1 and NaMT2 were from *Nicotiana attenuata*; OsMT1 was from the *Oryza sativa* Japonica Group; OsMT2 was from the *Oryza sativa* Indica Group; RcMT1 and RcMT2 were from *R. chinensis*; StMT was from *Solanum tuberosum*; TcMT1, TcMT2, TcMT3, and TcMT4 were from *Theobroma cacao*; ZmMT was from *Solanum Z. mays*.

The phylogenetic relationships of the four AcMTs were studied through the multiple sequence alignments of 34-aa MT domains (**Supplementary Table S3**) from 16 species. As depicted in **Figure 3**, the 34 MT genes could be classified into four groups (Type 1, Type 2, Type 3, and Type 4). However, the analysis revealed that the AcMTs were grouped in only Type 2 and Type 3, with no Type 1 and Type 4 noted. The phylogenetic relationships of different species were determined by constructing a phylogenetic tree. According to the phylogenetic tree, during evolution, the AcMT proteins diverged into two subgroups, Type 2 and Type 3, which remained conserved in kiwifruit.

### 3.3 Duplications and synteny analysis of the *AcMTs*

**Figure 4:**
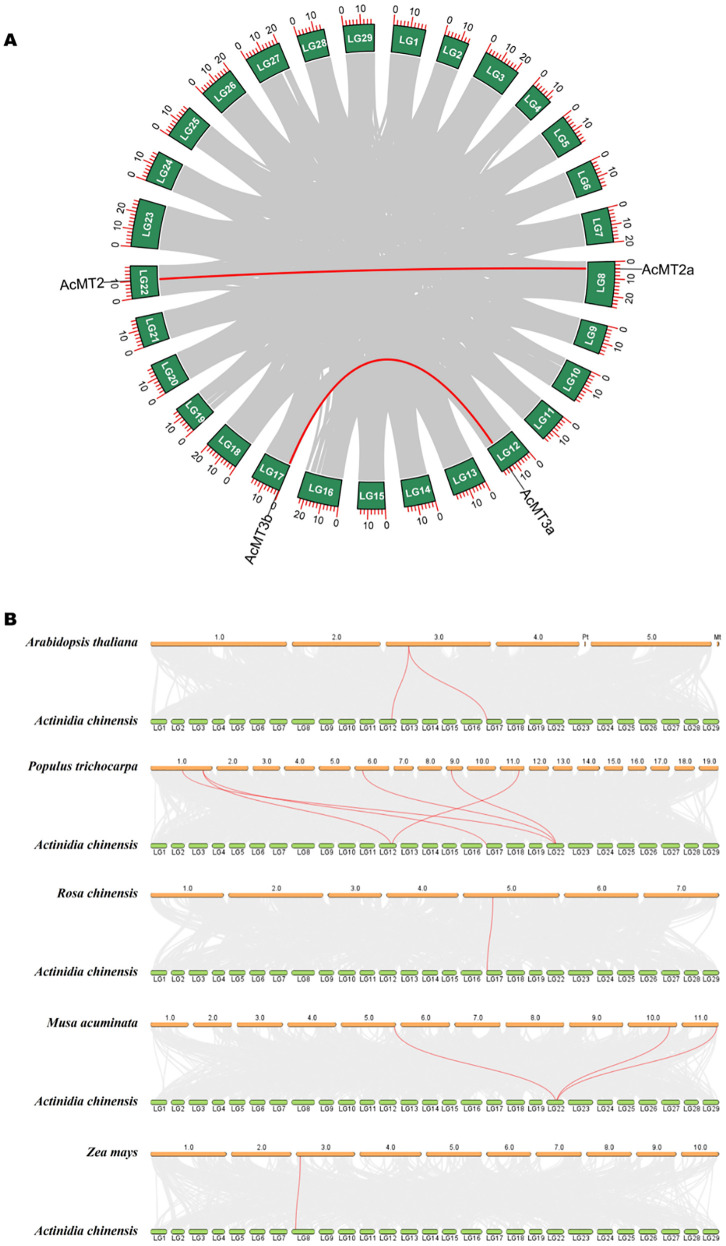
Duplication analysis and synteny analysis of the *AcMT* genes. **(A)** Duplication analysis of *MTs* in kiwifruit. Duplicate blocks are depicted using gray lines, while the duplicate *AcMT* gene pairs are depicted using red lines. **(B)** Syntenic maps between kiwifruit and other species. The syntenic *MT* gene pairs are depicted using red lines.

The collinear relationship analysis provides important insights into the functions and evolution of the *AcMT* families. As depicted in **Figure 4A**, two pairs (*AcMT2/AcMT2a* and *AcMT3a/AcMT3b*) of collinear relationships existed between the *AcMT* families. These two pairs of collinear relationships belonged to the segmental duplication of different chromosomes, suggesting the existence of segmental duplication between these *AcMT*s. The non-synonymous (*Ka*) and synonymous (*Ks*) values were utilized to evaluate the positive selection pressure of the duplication events. The *Ka*/*Ks* value of each duplicated *AcMT* pair was calculated (**Supplementary Table S4**), and the following were the indications: *Ka/Ks* = 1, neutral selection; *Ka/Ks* < 1, purifying selection; *Ka/Ks* > 1, positive selection (Gao et al., 2022). The *Ka*/*Ks* values of *AcMT2/AcMT2a* and *AcMT3a/AcMT3b* were 0.5093 and 1.2202, respectively. These values suggested that the evaluated *AcMT*s were subjected to purifying or positive selection pressure during evolution. Collectively, the above findings suggested that duplication events were the main reason for the amplification of *AcMTs*. In order to further explore the syntenic relationships of the AcMT families with other plant species, syntenic maps were constructed to explore the homology among *A. chinensis*, *A. thaliana*, *R. chinensis*, *Z. mays*, *M. acuminata*, and *P. trichocarpa* (**Figure 4B**). The results indicated that the four *AcMT*s had five pairs of MT collinearity with *P. trichocarpa*, three pairs of collinearity with *M. acuminata*, and one pair of collinearity with *A. thaliana*, *R. chinensis*, and *Z. mays* (**Supplementary Table S5**). The highest number of *AcMT* homologs was observed for *P. trichocarpa*, while *A. thaliana*, *R. chinensis*, and *Z. mays* presented the lowest number of homologs. These results demonstrated that the MT gene family members of different species might have originated from the same ancestor and performed a similar function.

### 3.4 *Cis*-elements predicted in the *AcMTs* promoters

**Figure 5:**
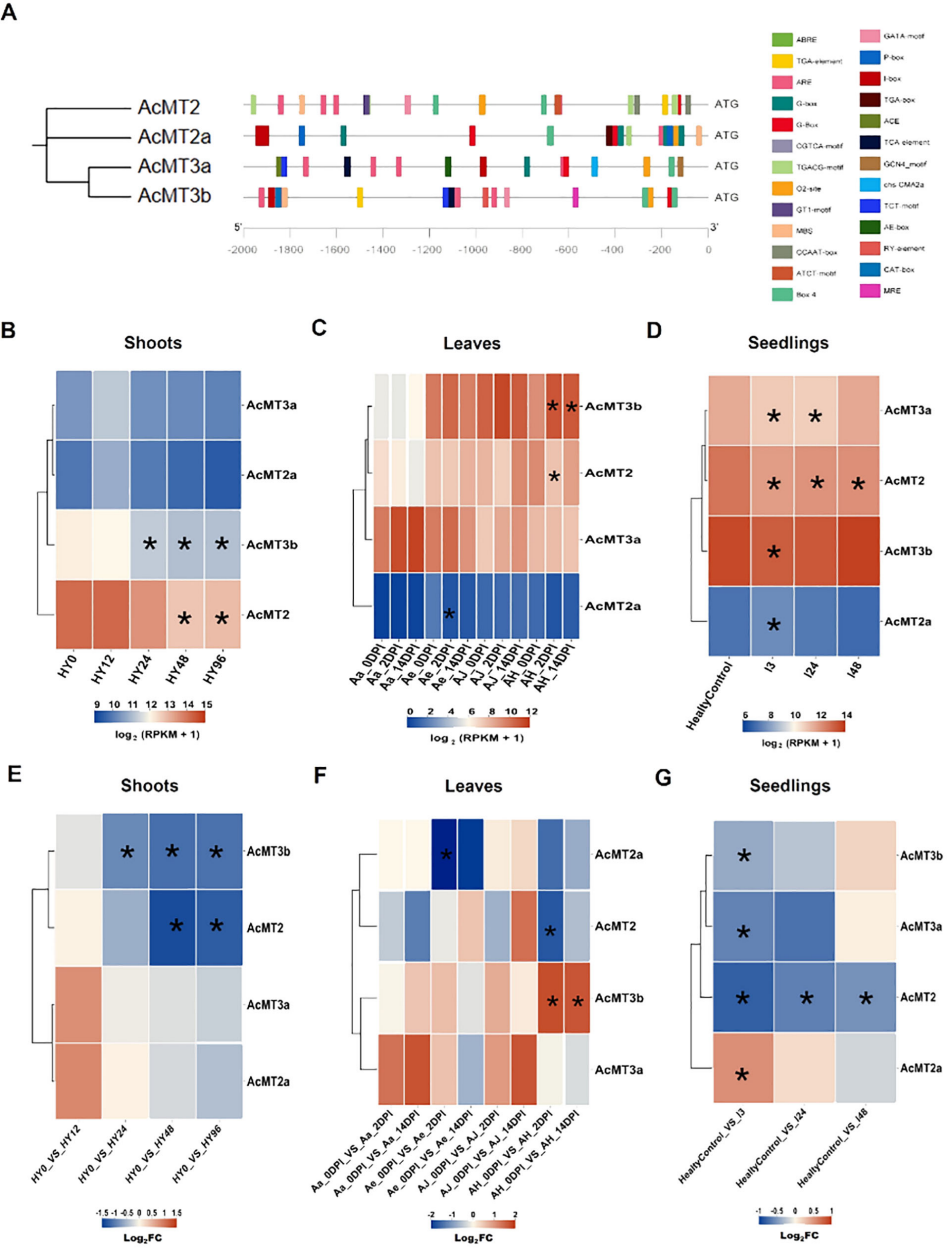
The analysis of the regulatory *cis*-elements and the expressions of *AcMTs* in response to Psa infection in four *Actinidia* species. **(A)** The predicted regulatory *cis*-elements in the promoters of the *AcMT*s in response to exposure to different plant hormones and stresses. **(B, E)** Heat maps generated at different time points for the levels of *AcMTs* in response to Psa infection in “Hongyang” shoot samples at levels of RPKM and log_2_(FC), respectively. HY0, HY12, HY24, HY48, and HY96 represent 0, 12, 24, 48, and 96 h post-Psa inoculation in HY. **(C, F)** Heat maps generated at different time points for the levels of *AcMTs* in response to Psa infection in the leaves of four *Actinidia* species/cultivars at levels of RPKM and log_2_(FC), respectively. AH, samples from the cultivar “Hongyang” of *A. chinensis*; AJ, samples from the cultivar “Jinyan” of *A. chinensis*; Ae, *Actinidia eriantha*; Aa, *Actinidia arguta*. 0DPI, 2DPI, and 14DPI were inoculated leaves for 0, 2, and 14 days, respectively. **(D, G)** Heat maps generated at different time points for the levels of *AcMTs* in response to Psa infection in the seedlings of *A. chinensis* var. *chinensis* at levels of RPKM and log_2_(FC), respectively. HealtyControl, healthy control plants; I3, I24, and I48 were inoculated plants for 3, 24 and 48 h post-inoculation, respectively. The gene expression levels were determined using the RPKM (expected number of reads per kilobase of the transcript sequence per million base pairs sequenced) method. The transcript levels are depicted using different colors on the scale, with blue and red indicating low and high expression levels, respectively. FDR cutoff value was set at 0.05, and asterisks in the middle of the heat maps meant points below the selected cutoff as significantly different expression. Asterisks in the middle of the heat maps meant points below the selected cutoff as significantly different expression.

The *cis*-elements within the promoter region of the *AcMTs* related to biotic and abiotic stresses were identified by obtaining and analyzing the upstream 2-kb genomic sequence prior to the start codon of each *AcMT* gene. The predicted *cis*-elements are depicted in **Figure 5A** and were observed to be associated with response to hormone and stress exposure (**Supplementary Table S6**). Five types of *cis*-elements were related to the plant response to hormone exposure: ABA response element, auxin response element, MeJA response element, GA_3_ response element, and SA response element. The remaining *cis*-elements were related to drought stress, anaerobic induction, light response, meristem expression, zein metabolism regulation, regulation of flavonoid biosynthetic genes, endosperm expression, and seed-specific regulation. These *cis*-elements were configured with different combinations within the *AcMT* promoter regions (**Figure 5A**).

### 3.5 Expression patterns of *AcMTs* in response to Psa infection

In order to determine the expression patterns of *AcMTs* in response to Psa infection, the RNA-seq data available in the Sequence Read Archive (SRA) database were analyzed for the expression of *AcMTs* in response to Psa infection in the shoots of “Hongyang” kiwifruit; the leaves of “Hongyang” kiwifruit, “Jinyan” kiwifruit, “Maohua” kiwifruit, and “Ruanzao” kiwifruit; and the seedlings of *A. chinensis* var. *chinensis* (**Supplementary Table S7**). The analysis of the RNA-seq dataset for the kiwifruit shoots with Psa inoculation (PRJNA514180) revealed (**Figures 5B–E**) that the expressions of *AcMT3b* and *AcMT2* were higher than those of the other two MTs, with a decreasing trend noted for the former two at the early stage (2 to 48 h) after Psa infection, followed by a recovery phase that began at 96 h. The transcriptional data (PRJNA328414) from the leaves of four kiwifruit varieties with Psa inoculation were analyzed. The results (**Figures 5C, F**) revealed that the regular expression of Type 2 MTs (*AcMT2* and *AcMT2a*) in the leaves of four kiwifruit varieties did not differ significantly after the Psa infection, while the Type 3 MTs (*AcMT3a* or *AcMT3b*) were upregulated in the leaves at 2 and 14 days after the Psa infection. In contrast, a sharp decline was noted in the expression of *AcMT3a* in the “Ruanzao” and “Maohua” kiwifruit and the expression of *AcMT3b* in “Maohua”, “Hongyang”, and “Jinyan” kiwifruit varieties. The analysis of the RNA-seq dataset for the Psa-inoculated kiwifruit seedlings (PRJNA436459) revealed (**Figures 5D, G**) that *AcMT2*, *AcMT3a*, and *AcMT3b* were downregulated during the early stage after the Psa infection, following which an increase was noted in the expressions of these MTs to the levels that were higher compared to the respective normal levels. The only exception to this was *AcMT2a*, the expression of which remained at a low level after the Psa infection.

### 3.6 Expression patterns of *AcMTs* in different tissues

**Figure 6:**
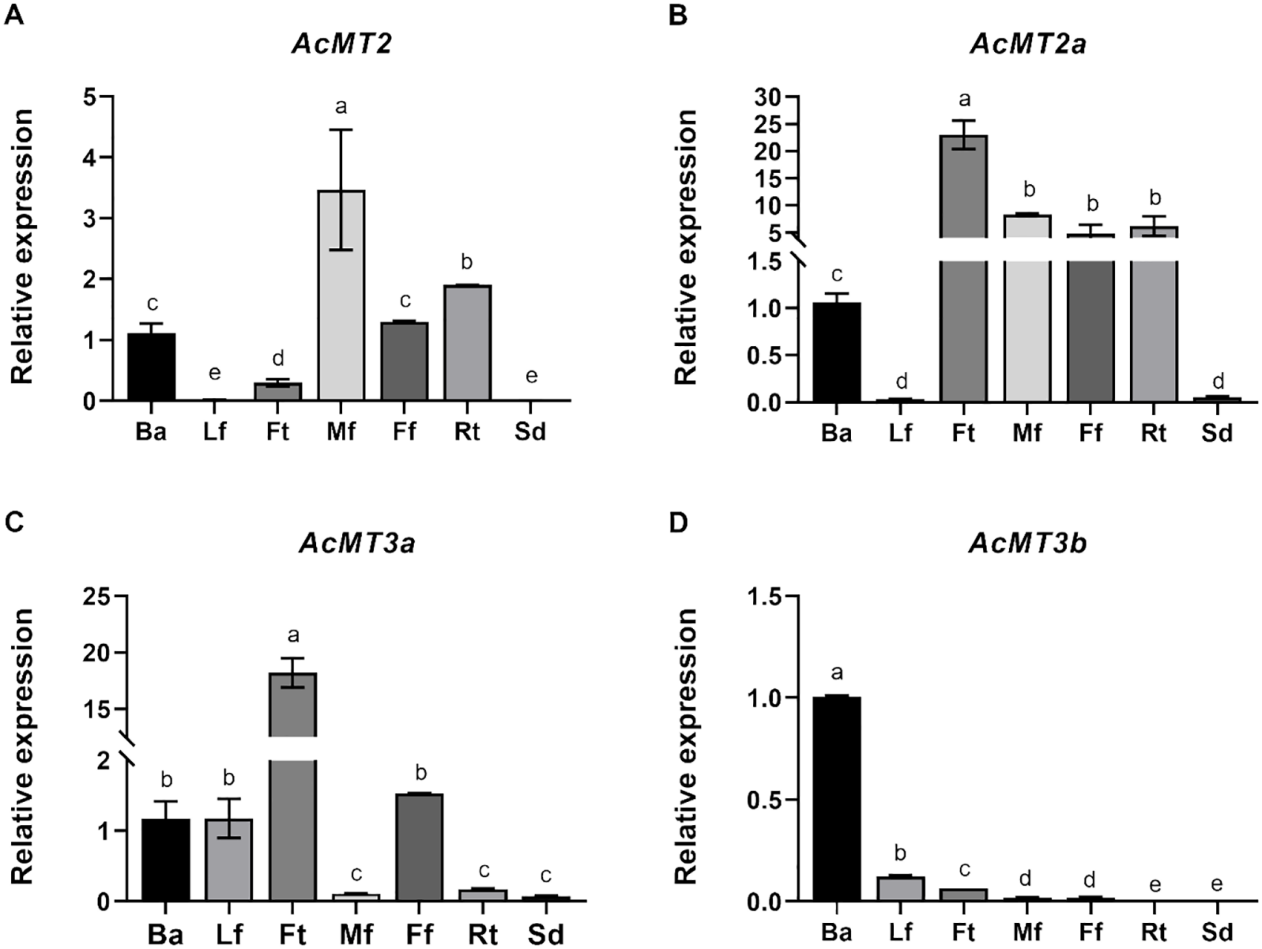
Expression patterns of the four *AcMT* genes in different tissues of kiwifruit. **(A)** *AcMT2*, **(B)** *AcMT2A*, **(C)** *AcMT3a*, and **(D)** *AcMT3b*. Ba, bark; Lf, leaf; Ff, Ft, fruit; Mf, male flower; Ff, female flower; Rt, root; Sd, seed. The values presented are means ± SEs of three independent experiments. Different letters indicate significant differences (*p* < 0.05) revealed in one-way analysis of variance.

The expression patterns of the four *AcMTs* in seven selected kiwifruit tissues, namely, bark, leaf, fruit, male flower, female flower, root, and seed, were determined. As depicted in **Figure 6**, the four *AcMT*s exhibited distinct expression patterns across the selected tissues. *AcMT2* was expressed at the highest level in the male flower, followed by root, female flower, bark, and fruit. The expressions of *AcMT2a* and *AcMT3a* were much higher in the fruit compared to the other tissues, while *AcMT3b* exhibited the highest expression level in the bark (**Figure 6D**).

### 3.7 Expressions of the four *AcMTs* in response to treatment with different plant hormones

**Figure 7:**
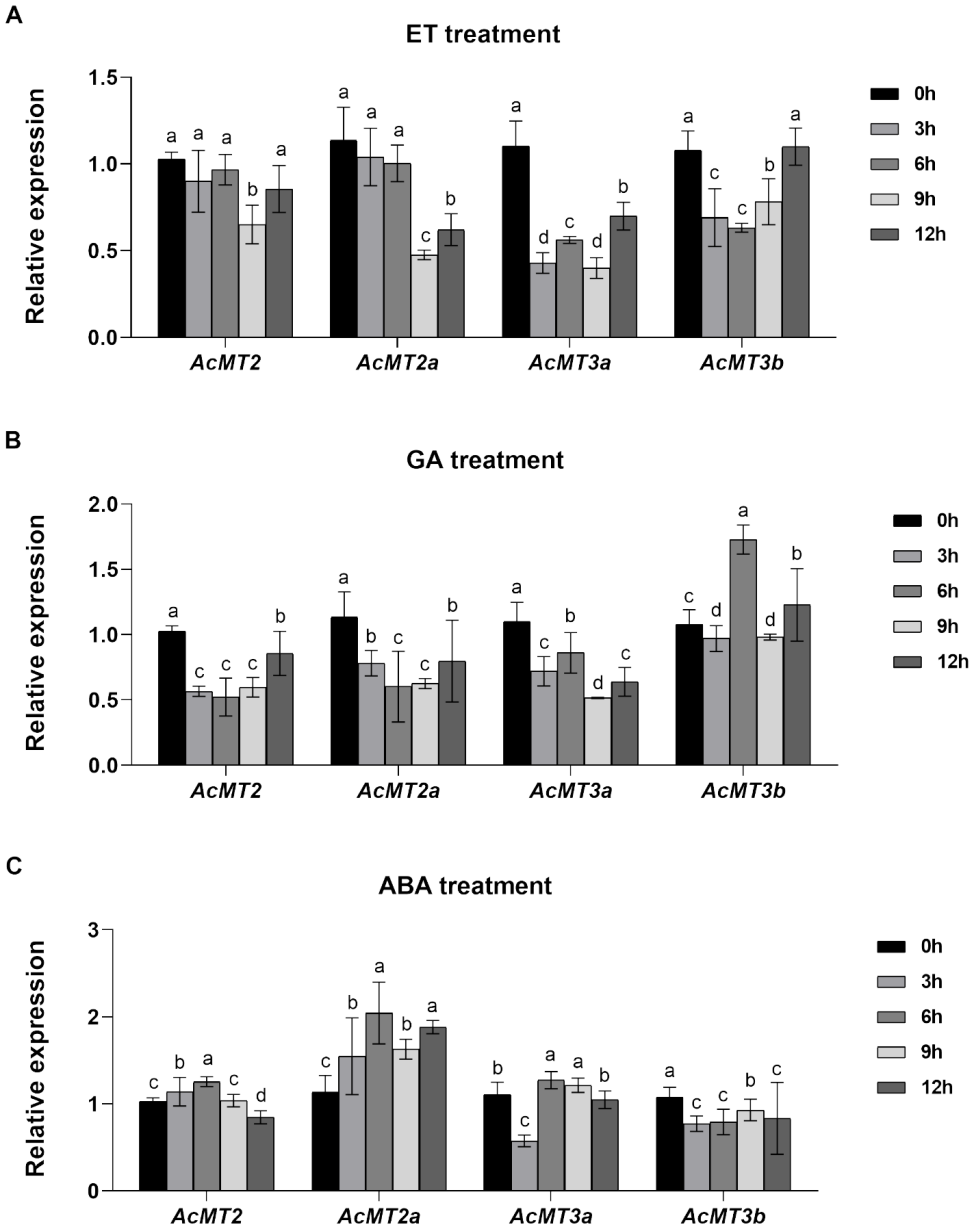
The effects of treatments with ET**(A)**, GA_3_ **(B)**, and ABA **(C)** on the expressions of the four *AcMTs* in the fruit determined through qPCR in kiwifruit. The values presented are means ± SEs of three independent biological replicates. Different letters indicate significant differences (*p* < 0.05) revealed in one-way analysis of variance.

The effects of treatment with different plant hormones on the expressions of the four *AcMT* genes were determined through qRT-PCR. The fruits of kiwifruit were treated with three plant hormones (ET, ethylene; GA_3_, gibberellic acid A3; ABA, abscisic acid) in this analysis (**Figure 7**). It was observed that ET treatment led to a decrease in the expressions of all *AcMTs* during the early stage after hormone exposure. The decrease in the expression of *AcMT2* was noted only at 9 h after treatment with ET. The expressions of the Type 3 MT genes (*AcMT3a* and *AcMT3b*) recovered to the baseline levels at 12 h (**Figure 7A**). Furthermore, as presented in **Figure 7B**, *AcMT2*, *AcMT2a*, and *AcMT3a* significantly downregulated after treatment with GA_3_. Notably, the expression of *AcMT3b* increased significantly and peaked at 6 h after treatment, followed by a decline to the baseline level. After treatment with ABA, the Type 2 MT genes (*AcMT2* and *AcMT2a*) exhibited significant upregulation, with the peak expression for each gene noted at 6 h, which was followed by a decline. At 12 h after treatment with ABA, the expression of *AcMT2* declined to a level lower than that noted at 0 h. In contrast, the Type 3 MT genes (*AcMT3a* and *AcMT3b*) were downregulated at 3 h. The expression of *AcMT3a* recovered to the baseline level at 12 h, while that of *AcMT3b* remained at a lower level compared to the baseline level until 12 h (**Figure 7C**).

### 3.8 Overexpression of AcMTs in *E. coli*

**Figure 8:**
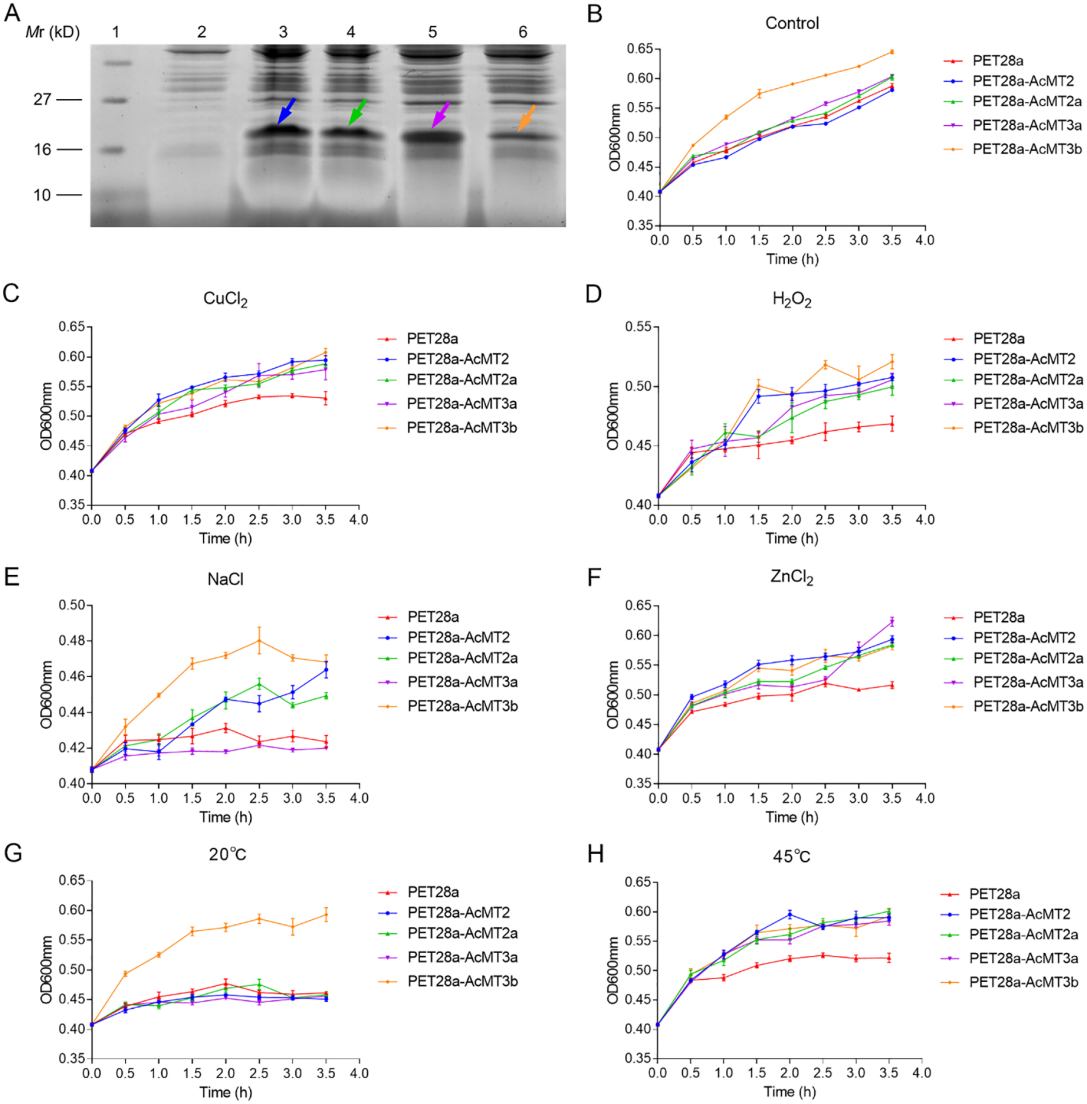
Effects of the overexpression of AcMTs on the growth of *E. coli* under heavy metal, H_2_O_2_, salt, and temperature stresses. **(A)** SDS-PAGE analysis of recombinant AcMT over-production in the *E. coli* cells harboring the blank vector pET-28a (Lane 2) or recombinant vectors pET-28a-AcMTs after IPTG induction (Lanes 3–6). Lane 1: Protein molecular weight marker; Lane 2: pET-28a- AcMT2; Lane 3: pET-28a-AcMT2; Lane 4: pET-28a-AcMT2a; Lane 5: pET-28a-AcMT3a; Lane 6: pET-28a-AcMT3b. Arrows indicate the respective tag peptide (His.Tag-thrombin recognition site-T7.Tag)–AcMT fusion proteins. **(B)** The relative populations of *E. coli* cells overexpressing different tag peptide–AcMT fusion proteins under culture in an LB medium without supplements. **(C)** The relative populations of *E. coli* cells overexpressing different tag peptide–AcMT fusion proteins under culture in the LB medium supplemented with 500 μM CuCl_2_ or 1 mM H_2_O_2_ **(D)**, 500 mM NaCl **(E)**, or 500 μM ZnCl_2_ **(F)**. **(G)** The relative populations of the *E. coli* cells overexpressing different tag peptide–AcMT fusion proteins under culture in the LB medium at 20°C or 45°C **(H)**. The results presented are the means ± SEs from three independent experiments.

In order to further explore the effects of the four AcMTs on the tolerance to heavy metals, temperature, and ROS, the recombinant pET28a-AcMTs fusion plasmid was overexpressed in *E. coli* (**Figure 8A**). It was observed that under normal growth conditions (LB broth, 37°C), the *E. coli* strain with AcMT3b overexpression exhibited the highest growth among all strains evaluated (**Figure 8B**). Moreover, under heavy metal stress (LB broth with 500 μM copper or zinc, 37°C) and high-temperature stress (LB broth, 45°C) conditions, the *E. coli* strains with overexpressed AcMTs exhibited better growth compared to the control with the overexpression of tag peptides (**Figures 8C, F, H**). The *E. coli* strains with *AcMT2* overexpression exhibited a higher growth ratio compared to the other strains upon treatment with 500 μM copper and zinc (**Figures 8C, F**). Furthermore, upon treatment with 1 mM H_2_O_2_ and 500 mM NaCl, the growth of all strains was greatly repressed, although the *E. coli* strain with AcMT3b overexpression exhibited better growth compared to the other strains (**Figures 8D, E**). It was also observed that none of the recombinant HbMT proteins (except for AcMT3b) were advantageous to *E. coli* growth under low-temperature (20°C) conditions (**Figure 8G**).

## 4 Discussion

The levels of *MT* genes in a given plant species are dependent on the genomic ploidy of the species, regardless of whether it is a dicot or monocot (Huang et al., 2018; Gao et al., 2022). In the present study, four MT members were identified in “Hongyang” kiwifruit, including two Type 2 MTs (AcMT2 and AcMT2a) and two Type 3 MTs (AcMT3a and AcMT3b), while no Type 1 and Type 4 MTs were detected (**Figure 1B**) (Cobbett and Goldsbrough, 2002). Similarly, previous studies have identified certain plant species lacking one or two types of MTs, such as rubber tree (without Type 1 and Type 4), *Phoenix dactylifera* (without Type 1 and Type 3), and *Medicago truncatula* (without Type 4) (Huang et al., 2018). The lack of Type 1 and Type 4 MTs in “Hongyang” kiwifruit could be attributed to the functional complementarity and redundancy among different types of MTs during the evolution of kiwifruit.

The number and distribution of cysteine residues in the N-terminus and C-terminus regions of MTs are known to be conserved and related to the function of these MTs. In the present study, AcMT3a and AcMT3b presented cysteine residue characteristics similar to those of the other Type 3 MTs, with four Cys located at the N-terminal region and six Cys located at the C-terminal region. AcMT2 and AcMT2a had the Cys characteristics of other Type2 MTs, with three Cys-X-Cys motifs at the C-terminus of their sequences. Meanwhile, AcMT2a presented a unique structure with eight Cys residues (CCXXXCXCXXXCXCXXXCXXCXXX) at the N-terminus of its structure. AcMT2a was, therefore, regarded as an atypical MT. In previous studies, the Cys residues in MTs were demonstrated to affect their heavy metal-chelating function (De Oliveira et al., 2020). In addition, mutations in the Cys residues of NtMT2F were reported to significantly reduce the tolerance of *A. thaliana* to heavy metals (Li et al., 2023). Cysteine substitution with tyrosine (C3Y) in PtMT2b reportedly enhanced the Cd tolerance in the transformed yeast (De Oliveira et al., 2020). The missing Cys residues at the C-terminus of ScMT10 could enhance the freezing tolerance in the heterogeneously expressed tobacco plants (Feng et al., 2022). Similarly, in the present study, the bacterial strain overexpressing AcMT2 exhibited faster growth compared to the bacterial strains overexpressing AcMT2a under Cu and Zn stresses. These results indicated that the substitution of Cys residues in AcMT2a significantly reduced its ability to chelate heavy metals. Further studies should, therefore, be conducted to elucidate the role of Cys residues in MTs in influencing tolerance to heavy metals in plants.

An increasing number of studies have recently reported that the same types of plant MT genes exhibit varied expression patterns in different plant tissues. *HbMT2a*, for instance, was reported to exhibit a flower-specific expression (Huang et al., 2018), while *NtMT2F* is primarily expressed in roots and old leaves, according to reports (Yu et al., 2021). *CsMT2*, on the other hand, exhibits a relatively universal expression in different tissues (Zhou et al., 2019). In the present study, *AcMT2* and *AcMT2a* exhibited the highest expression levels in the fruit, followed by male flower, female flower, root, and bark, although both MT genes were expressed in low levels in the leaf and seed. Meanwhile, *AcMT3a*, similar to the other members of Type 3 *MT* genes, exhibited the highest expression in the fruit, while *AcMT3b* exhibited the highest accumulation of its transcripts in the bark. It is noteworthy that the highest expression of *AcMT2* was noted in the male flower, the highest expressions of *AcMT2a* and *AcMT3a* were noted in the fruit, and the highest expression of *AcMT3b* was noted in the bark, indicating that *AcMTs* have discrete and overlapping roles in different tissues. The high expression of AcMT2 in the roots might be a contributor to the enhanced resistance of plants to heavy metal exposure, while the high expression of AcMT3b in the bark could serve as a barrier against cold stress and pathogen invasion, although further studies are warranted to confirm these hypotheses.

The bacterial canker caused by Psa has been detected in the main kiwifruit-producing nations since 2007 (Vanneste et al., 2011; Chapman et al., 2012). The rapid transmission and strong pathogenicity of Psa have led to serious production and economic losses in several nations, causing Psa to become a major limiting factor in the development of the kiwifruit industry (Mazzaglia et al., 2012; Vanneste, 2012). Certain genes involved in the plant’s response to Psa infection have been characterized in previous studies, including the genes encoding pentatricopeptide repeat (PPR) proteins. These PPR genes are thought to regulate plant–pathogen interactions by controlling the degree of RNA editing, particularly the composition of the NDH complex (Zhang et al., 2023). According to previous studies, the MTs in plants play protective roles against pathogen attacks (Reymond et al., 2000; Wong et al., 2004). In the present study, the expression levels of the main *AcMTs* (with a higher expression level compared to the other MTs) in each tissue of the kiwifruit were revealed to be downregulated during the early stage after Psa infection, followed by a recovery phase, indicating that different combinations of AcMTs serve as the main barriers against Psa infection in different tissues of the kiwifruits. These findings were similar to those reported previously for the leucine-rich repeat receptor-like proteins (LRR-RLPs) in kiwifruit (Cao et al., 2024). Previous studies have also demonstrated that while ROS accumulation causes severe cellular damage through protein oxidation, lipid peroxidation, nucleic acid damage, and enzyme inhibition (Sharma et al., 2012), it plays a central role in plant defense against pathogens by limiting the spread of pathogenic bacteria (Lamb and Dixon, 1997). The regulation of ROS levels is considered important for plant disease resistance. All *AcMTs* evaluated in the present study exhibited ROS-scavenging abilities, as evidenced by the significant tolerance of the *E. coli* strain with overexpression of each *AcMT* to the ROS stress. The downregulation of the main *AcMTs* in each tissue during the early stage after Psa infection might be beneficial to the ROS accumulation in the plant for acting against the pathogen invasion. Once the ROS levels are too high to cause oxidative damage, the expression levels of the main *AcMTs* in each tissue begin to return to the baseline levels, resulting in the observed recovery phase.

MTs are involved in numerous physiological processes, such as cell growth proliferation and regulation, plant growth and defense against pathogens, homeostasis, and heavy metal toxicity protection (Khalid et al., 2020). These functions of MTs are similar to those of the other functional genes involved in plant growth and stress resistance in kiwifruit, which are reportedly regulated precisely through plant hormone-related signaling pathways. *CsMT4* (Zhou et al., 2019) and *BrMT1* (Ahn et al., 2012), for example, are reported to exhibit increased levels upon ABA treatment, conferring the plants with the ability to withstand osmotic pressure and drought resistance. *HbMT2b* levels (Huang et al., 2018) were markedly depressed upon ethephon treatment in a previous study, which increased the risk of oxidative stress in tapped rubber trees. In the present study, ET and GA_3_ significantly downregulated most of the *AcMT* genes, indicating that during fruit ripening or stages of rapid plant growth, kiwifruits were more susceptible to damage due to external stress when the levels of ET and GA_3_ were higher. In contrast, when the synthesis of ABA increased, causing the plant growth to be inhibited and the plant to enter into the dormancy stage, the synthesis of Type 2 MTs (*AcMT2* and *AcMT2a*) was probably increased to enhance the ability of kiwifruit to withstand the external stresses.

## 5 Conclusions

In the present study, four *AcMTs* were identified in kiwifruit, and their sequence characteristics were determined. The expression profiles of these four *AcMTs* in response to Psa infection and treatment with plant hormones were determined, and these profiles implied the roles of these AcMTs in plant defense against pathogens. Furthermore, the *E. coli* strains overexpressing the AcMTs exhibited better growth under H_2_O_2_, heavy metal, and extreme temperature stresses, indicating that these AcMTs could enhance the tolerance of the bacteria to these stresses. These findings provide insights into the potential functions of the *MT* genes in kiwifruits, and the information would also be useful for elucidating the mechanism underlying stress tolerance in kiwifruits.

## Data Availability

The original contributions presented in the study are included in the article/**Supplementary Material**. Further inquiries can be directed to the corresponding author.
