# Supplementary figures and images for: Metallothionein family genes in kiwifruit: characterization and determining their roles in plant’s response to different stresses
Source: Front Plant Sci. 2024 Oct 18;15:1425072. doi: 10.3389/fpls.2024.1425072 (PMC11529040; doi:10.3389/fpls.2024.1425072)

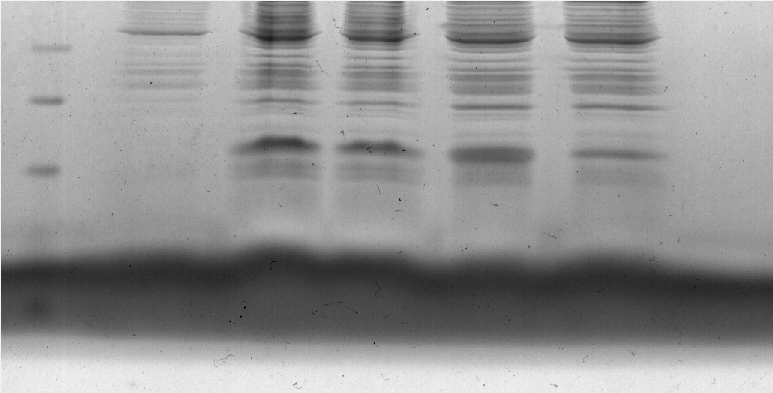

Supplement: Supplementary file 1 [file DataSheet1.zip › Gel Image/Gel Image 1.tif]

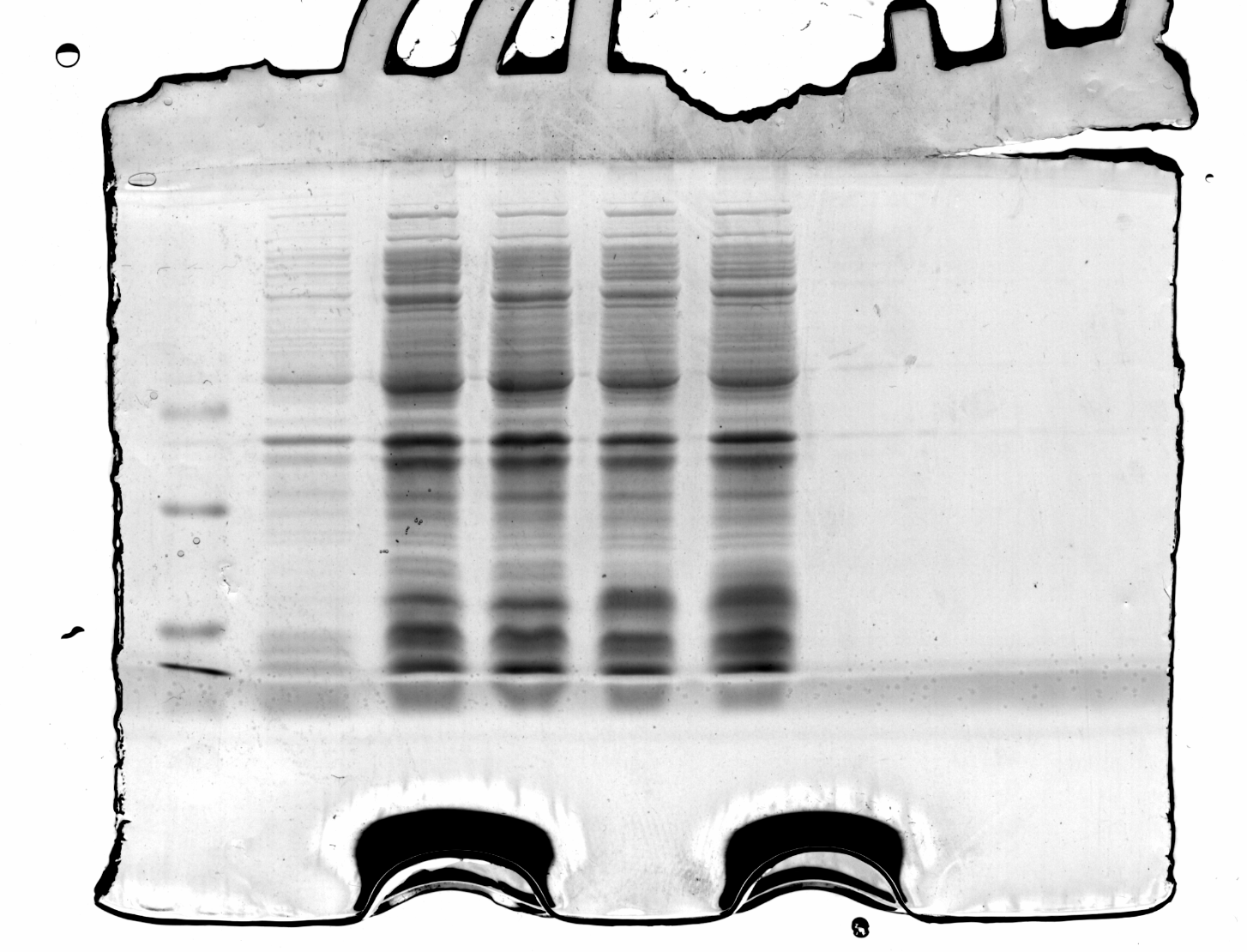

Supplement: Supplementary file 1 [file DataSheet1.zip › Gel Image/Gel Image 2.tif]

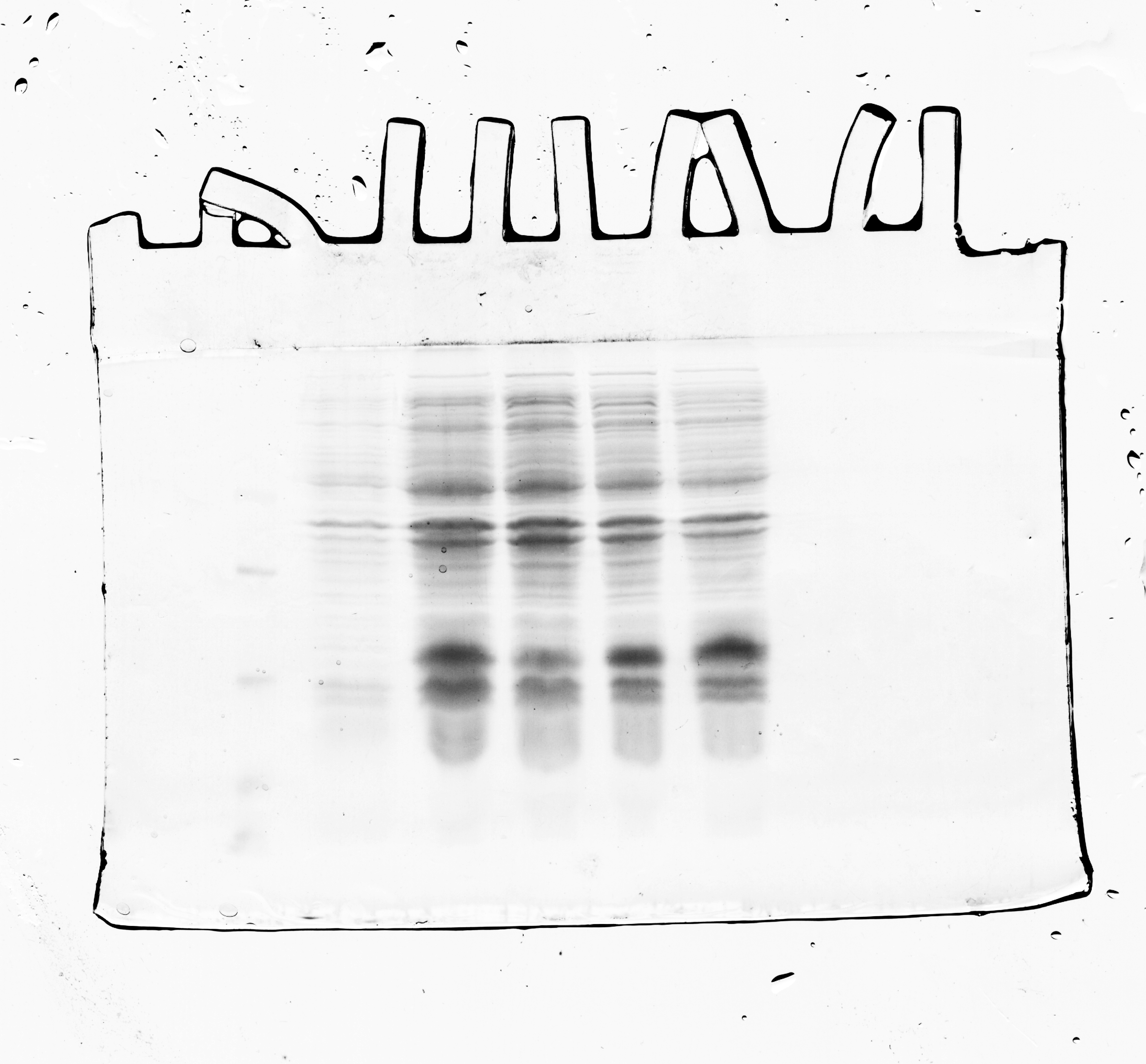

Supplement: Supplementary file 1 [file DataSheet1.zip › Gel Image/Gel Image 3.tif]
